# NEWS

**DOI:** 10.7189/jogh.02.010202

**Published:** 2012-06

## THE BILL AND MELINDA GATES FOUNDATION

• Bihar Chief Minister Nitish Kumar was chosen by the Bill and Melinda Gates Foundation for its first Gates Vaccine Innovation Award for improving routine immunisation from 18.6% in 2005 to 70% in 2011. (*India.com*, 03 Jan 2012)

• Bill Gates donated US$ 750 million to the struggling Global Fund to Fight AIDS, Tuberculosis and Malaria in January 2012. The donation was made as a promissory note intended to tide the fund over. Saudi Arabia recently made a contribution to the fund, and Brazil has followed Russia's lead among emerging nations in saying that it needed no further support for some health programmes. (*New York Times*, 26 Jan 2011)

• The BMGF, government aid agencies, WHO, and 13 drug companies pledged nearly US$ 800 million and an increased drug supply toward a new push to wipe out 10 tropical diseases by 2020. The diseases, from leprosy to river blindness, affect 1.4 billion people worldwide. The companies have agreed even to open up their compound libraries - potential drug treatments that have gone through some tests – to the public-private partnership Drugs for Neglected Diseases Initiative (DNDi). (*Wall Street Journal*, 30 Jan 2012)

• The Bill & Melinda Gates Foundation announced US$ 7.7 million in funding for 10 new grants to identify biomarkers for diagnosing tuberculosis (TB) in low-resource settings. This new grant program, Biomarkers for the Diagnosis of Tuberculosis, supports innovative research into TB biomarkers to facilitate the development of a simple low-cost tool that can quickly and accurately diagnose TB in low-resource settings. (*BMGF*, 09 Feb 2012)

• Dr Cyrus Poonawalla of the Serum Institute in Hadapsar, India, revealed that the Bill & Melinda Gates Foundation may tie up with the Serum Institute to develop injectable polio vaccines. At present, The Global Alliance for Vaccine and Immunisation, Gates foundation and the Serum Institute are partners in several immunisation programmes around the world. The institute exports vaccines for GAVI projects, while the Gates Foundation funds clinical trials, Poonawalla said. (*Times of India*, 02 June 2012)

## THE GAVI ALLIANCE

• The GAVI Alliance recently decided to add HPV vaccines to its list of vaccines subsidized in the poorest countries, thanks in part to tiered pricing by the manufacturers; now it is feasible to seriously consider widespread sustained vaccination of populations in low-resource settings who do not have access to care (*Cancer Prevention Research*, 05 Jan 2012)

• The GAVI Alliance said that as much as US$ 6.7 million intended for buying vaccines for children in poor countries was either stolen or misused. In Cameroon, as much as US$ 4.2 million was misused between 2007 and 2010, including US$ 1.8 million that is being investigated for theft, the GAVI Alliance announced in January 2012. As much as US$ 2.5 million was misused in Niger, of which US$ 1.5 million may have been stolen, the Geneva-based organization said. (*Bloomberg*, 19 Jan 2012)

• US President Barack Obama and UK Prime Minister David Cameron have cited GAVI as a highly cost-effective, life-saving investment. At a joint press conference at the White House, the two leaders referred to last year's landmark pledging conference in London, where donors committed U$S 4.3 billion to help GAVI and its partners immunise an additional 250 million children and avert an estimated four million future deaths by 2015. (*GAVI Alliance*, 20 March 2012)

• The GAVI Alliance has secured new prices with manufacturers for vaccines against rotavirus that are 67% lower than before. The bulk (95%) of the vaccines contracted – 132 million doses – will be procured at a cost of US$ 5 per (two dose) course instead of US$ 15, while the same course costs US$ 177 in the US. The deal will enable the Alliance to provide the vaccines to eight developing countries this year for some three million children. (*BMJ*, 11 Apr 2012)

• GAVI Board Chair Dagfinn Høybråten said the appointment of three women to the GAVI Alliance Board “achieves a key target on gender”. Her Royal Highness the Infanta Cristina of Spain, Dr Maria C. Freire, and Yifei Li took up their positions with immediate effect, resulting in 11 out of 26 Board members being women. In addition, Suraya Dalil, who has been approved by the Afghan Parliament as the new Minister of Public Health, is also a GAVI board member as of January 2012. GAVI’s Board achieved its target of at least 40% representation for both genders within two years of approving guidelines at its meeting in July 2010. (*Center for Vaccine Ethics Policy*, 14 Apr 2012)

## THE WORLD BANK

• A free software program created by the World Bank researchers should assist staff and policy-makers in evidence-based decision making. The free software is especially valuable for analysts in developing countries. With built-in modern statistical technology and a user-friendly interface, ADePT empowers policy practitioners to conduct sophisticated economic analysis. It also serves as a platform for researchers to share findings. (*The World Bank*, 05 Jan 2012)

• The World Bank has warned developing countries they need to be prepared for shocks as global economic growth slows. The organisation is now predicting a 0.3% contraction for the eurozone in 2012. The World Bank chief economist, Justin Yifu Lin, said that “...developing countries need to evaluate their vulnerabilities and prepare for further shocks, while there is still time”. The World Bank is predicting growth of 5.4% for developing countries in 2012 and 1.4% for high-income countries, down from its forecasts of 6.2% and 2.7% respectively in June 2011. (*BBC*, 18 Jan 2012)

• The World Bank is launching an innovative lending instrument that ties funding directly to the delivery of results. “Program-for-Results” will not provide financing to cover a program's expense. Instead, it will disburse money upon the delivery of predefined results. The World Bank is expected to channel 5% of its total portfolio through PforR. The CGD said this equates to some US$ 1 billion, or 20 projects per year, and the instrument will be assessed after the first two years. (*Devex*, 24 Jan 2012)

• Ministers of Finance from the BRICS group of emerging market powerhouses – Brazil, Russia, India, China and South Africa – met on the sidelines of a G20 meeting in Mexico City and agreed the top World Bank job should be open to all countries. They agreed to reject the tradition that an American would automatically be selected to head the World Bank, and they will look at putting forward their own candidate for the open job. US President Barack Obama, however, nominated a Korean-American known for his work in fighting disease in impoverished countries. Jim Yong Kim, 52, is president of Dartmouth College, the former director of the Department of HIV/AIDS at the World Health Organization, and a founder of Partners in Health. He was appointed in mid-April. (*New York Times*, 16 Apr 2012)

• Years after debt campaigners succeeded in persuading the International Monetary Fund (IMF), World Bank and G8 to abolish debts worth billions of dollars owed by developing countries, figures show total external debts are once again on the increase. Data in the World Bank's Global Development Finance 2012 report shows total external debt stocks owed by developing countries increased by US$ 437 billion over 12 months, to stand at US$ 4 trillion at the end of 2010, the latest period for which data are available. (*The Guardian*, 15 May 2012)

## UNITED NATIONS (UN)

• In its annual report on global employment, the United Nations International Labour Organization (ILO) said that the world needs to create 600 million new jobs over the next decade to sustain economic growth and maintain social stability. The report, entitled 'Global Employment Trends 2012: Preventing a deeper jobs crisis', states that the world faces the additional challenge of creating decent jobs for the estimated 900 million workers who subsist on less than US$ 2 a day. (*UN News*, 23 Jan 2012)

• Cases of dementia – and the heavy social and financial burdens associated with them – are set to soar in the coming decades as life expectancy and medical care improve in poorer countries, the UN’s health agency (WHO) said in its World Dementia Report. Some 35.6 million people were living with dementia in 2010, but that figure is set to double to 65.7 million by 2030. “The numbers are already large and are increasing rather rapidly”, said Dr Shekhar Saxena, the head of WHO's mental health division. (*Associated Press*, 11 Apr 2012)

• The global campaign to fight malaria is appealing for US$ 3.2 billion to try to reach the UN goal of “near-zero” deaths from this mosquito-borne disease by the year 2015. There has been “great progress” in reducing malaria deaths using bed nets, insecticide spray and drugs, said Ray Chambers, the UN secretary-generals special envoy for malaria. (*Washington Post*, 24 Apr 2012)

• A new United Nations initiative seeks to involve private businesses in helping developing countries tackle corruption and strengthen their ability to fight it. “Corruption has a disproportionate impact on poor communities and a corrosive effect on the fabric of societies across the globe”, the Executive Director of the UN Office on Drugs and Crime, Yury Fedotov, said at the sidelines of the 21st Session of the Commission on Crime Prevention and Criminal Justice in Vienna, Austria. (*UN News*, 24 Apr 2012)

• UN chief Ban Ki-moon declared in May 2012 that Britain's Prime Minister David Cameron, President Susilo Bambang Yudhoyono of Indonesia and Liberia's President Ellen Johnson-Sirleaf will lead a global panel to set international targets on sustainable development. The three leaders will represent the world's rich, middle- and low-income countries, and start their work after a major summit in Rio de Janeiro in June. (*AFP*, 09 May 2012)

## UN AIDS

• South Africa is home to the highest number of HIV cases in the world. However, Sheila Tlou, UNAIDS regional director for East and Southern Africa, said that the country should see a massive reduction by the end of the decade after a sea-change in government policy. South Africa now has more people with HIV infections than any country in the world, with 5.6 million, because of a lack of political commitment in the past. However, Ms Tlou said that “...there is a turnaround in the new government under President Jacob Zuma, which is committed, and by 2020 there will be massive reductions in South Africa”. (*AFP*, 19 Jan 2012)

• The Russian State Research Center for Virology and Biotechnology, Vektor, has successfully completed the first stage of clinical trials of an HIV vaccine. The vaccine induces a strong antibody (antigen), as well as cellular response, said Vektor’s director Alexander Sergeyev. The vaccine is now to be approved for the second stage of tests. (*RIA Novosti*, 06 Feb 2012)

• Cuba's top biotech teams have successfully tested a new AIDS vaccine on mice, and are ready to soon begin human testing. At the bioconference in Havana, it has been announced that the vaccine TERAVAC-HIV-1 was made from recombinant proteins aiming “to cause a cellular response against the (HIV) virus”. (*AFP*, 06 Mar 2012)

• The United Nations Secretary-General Ban Ki-moon has issued his first report on HIV to the UN General Assembly since the 2011 High Level Meeting on AIDS. In the report, he highlighted “…the urgent need to achieve immediate, tangible results and for the AIDS response to be smarter, more strategic, more efficient, and grounded in human rights”. (*UNAIDS*, 30 Apr 2012)

• A recent study that evaluated US aid program aimed at helping foreign countries battle the AIDS epidemic showed that about 740 000 lives were saved from 2004–2008. The US President's Emergency Plan for AIDS Relief, or PEPFAR, was started by former president George W. Bush in 2003 with a five-year, US$ 15 billion investment in global AIDS in 15 countries. (*AFP*, 15 May 2012)

## UNICEF

• UNICEF is preparing ambitious plans to update, strengthen and vastly expand its global vaccination programme (*UNICEF*, 03 Jan 2012)

• According to the Humanitarian Action for Children 2012 report, launched at the end of January 2012, the ongoing crisis in the Horn of Africa will remain a significant part of UNICEF's global humanitarian response in the coming year. UNICEF asked for US$ 1.28 billion to meet the needs of the most vulnerable children and their families in 25 countries and territories – a 9% decrease from last year's appeal. (*UNICEF*, 26 Jan 2012)

• UNICEF announced that it had joined the International Aid Transparency Initiative (IATI) to improve public accessibility to information on how aid is spent. UNICEF joints the World Bank, Britain’s Department for International Development, the African Development Bank, the European Commission and others in the initiative. By creating common standards for sharing information about aid, IATI will make that information much more accessible to all. As part of its commitment to greater transparency and accountability, UNICEF will make public online the volume, allocation and results of development expenditure. (*UNICEF*, 13 Apr 2012)

• The first-ever World Immunization Week took place from 21–28 April 2012. UNICEF offices around the world are engaging in immunization campaigns and raising awareness about the importance of vaccines to child survival; UNICEF is the world's largest buyer of vaccines for the world's poorest countries, and has been supplying vaccines to children for over 50 years. (*UNICEF*, 24 Apr 2012)

• A study commissioned by UNICEF in January 2012 showed that 46% of the 600 respondents participating in a baseline Knowledge, Attitude and Practices (KAP) study are ignorant about vaccination being a way to prevent polio, while only one in three parents is concerned that their child is at risk of contracting polio this year. (*The News/Pakistan*, 05 June 2012)

## WORLD HEALTH ORGANIZATION (WHO)

• The World Health Organization Executive Board agreed to propose to the World Health Assembly the establishment of a mechanism for international collaboration on counterfeit and substandard medical products, but with the exclusion of trade and intellectual property issues. One of the contentious issues around counterfeit drugs has been the suspicion on the part of some developing countries that concerns about counterfeit and substandard medicines are being purposely confused with trade in legitimate generic medicines from those countries. Removing intellectual property and trade from WHO discussions likely minimises the possibility of confusion. (*IP–Watch*, 28 Jan 2012)

• Organisations working to combat non-communicable diseases worldwide say that the timetable for developing crucial targets “cannot afford to slip”. Ms Judith Watt, interim director of the Non-Communicable Disease Alliance, which represents non-governmental organisations and other civil groups, said it was vital that the World Health Organization fulfil its commitments this year to recommending voluntary global targets for the prevention and control of non-communicable diseases. (*BMJ*, 30 Jan 2012)

• On World Health Day (7 April), WHO is calling for urgent action to ensure that people reach old age in the best possible health. In the next few years there will be more people in the world aged over 60 than children aged less than five, for the first time in human history. By 2050, 80% of the world's older people will be living in low- and middle-income countries. (*WHO*, 03 Apr 2012)

• The World Health Organisation (WHO) has warned that a third of the world's population is carrying tuberculosis, and the disease could become incurable if governments fail to act. Lack of funding for public health programmes, the sale of inaccurate blood tests, and the misuse of drugs, particularly in the private health sector, are hampering the fight against the disease and leading to drug resistance. (*The Independent*, 13 May 2012)

• The World Health Organization nominated its current chief Margaret Chan for a second term at the Director General of the WHO. Ms Chan was the only candidate put forward to the WHO's executive board, who made the official nomination at a meeting in Geneva. She was re-elected in May 2012, at the World Health Assembly (*Associated Press*, 25 May 2012)

